# Digital financial inclusion, population structure, and consumption upgrades: Evidence from China

**DOI:** 10.1371/journal.pone.0316823

**Published:** 2025-01-22

**Authors:** Qianwen Liu, Jianjie Zheng, Shihui Luo

**Affiliations:** 1 Faculty of Economics, Guangxi University, Nanning, Guangxi, China; 2 School of Applied Economics, Guangdong Baiyun University, Guangzhou, Guangdong, China; Chongqing Technology & Business University, XX

## Abstract

This study empirically tests the role of population structure in the process of digital financial inclusion empowering the upgrading of consumption structure by taking 248 prefecture-level cities between 2013 and 2019 as the research objects. The results of this study are as follows. First, digital financial inclusion will expand the scale of consumption. Second, digital financial inclusion can promote the upgrading of consumption structure. Third, population structure will regulate the promotional effect of digital financial inclusion on the upgrading of consumption structure. Specifically, child dependency ratio has a positive moderating effect on digital financial inclusion-driven the upgrading of consumption structure. Moreover, sex ratio has a positive moderating effect on digital financial inclusion-driven the upgrading of consumption structure. Therefore, it is suggested that vigorously promote and develop digital financial inclusion, implement and improve the three-child policy, and expand the elderly consumer industry to better promote consumption upgrading.

## 1. Introduction

Digital financial inclusion is a process that ensures vulnerable groups access financial services at affordable costs, providing avenues for savings, investment, consumption, and insurance for those who previously lacked bank accounts [1,2]. With the rapid advancement of information technology, digital financial inclusion, as an innovative financial service model, has been widely promoted and implemented globally [3,4]. It effectively lowers the barriers to access financial services and significantly improves transaction efficiency, successfully providing the general public with more convenient and efficient financial solutions [5]. Research on digital financial inclusion covers multiple aspects including government policies, social welfare, rural revitalization, corporate performance and green economy, demonstrating its significance and widespread application in promoting sustainable economic, social, and environmental development [6–10]. Furthermore, digital financial inclusion effectively reduces financing costs for SMEs and enhances their external financing environment, while promoting the redirection of regional capital from the fictitious to the substantial economy [11,12].

**Fig 1:**
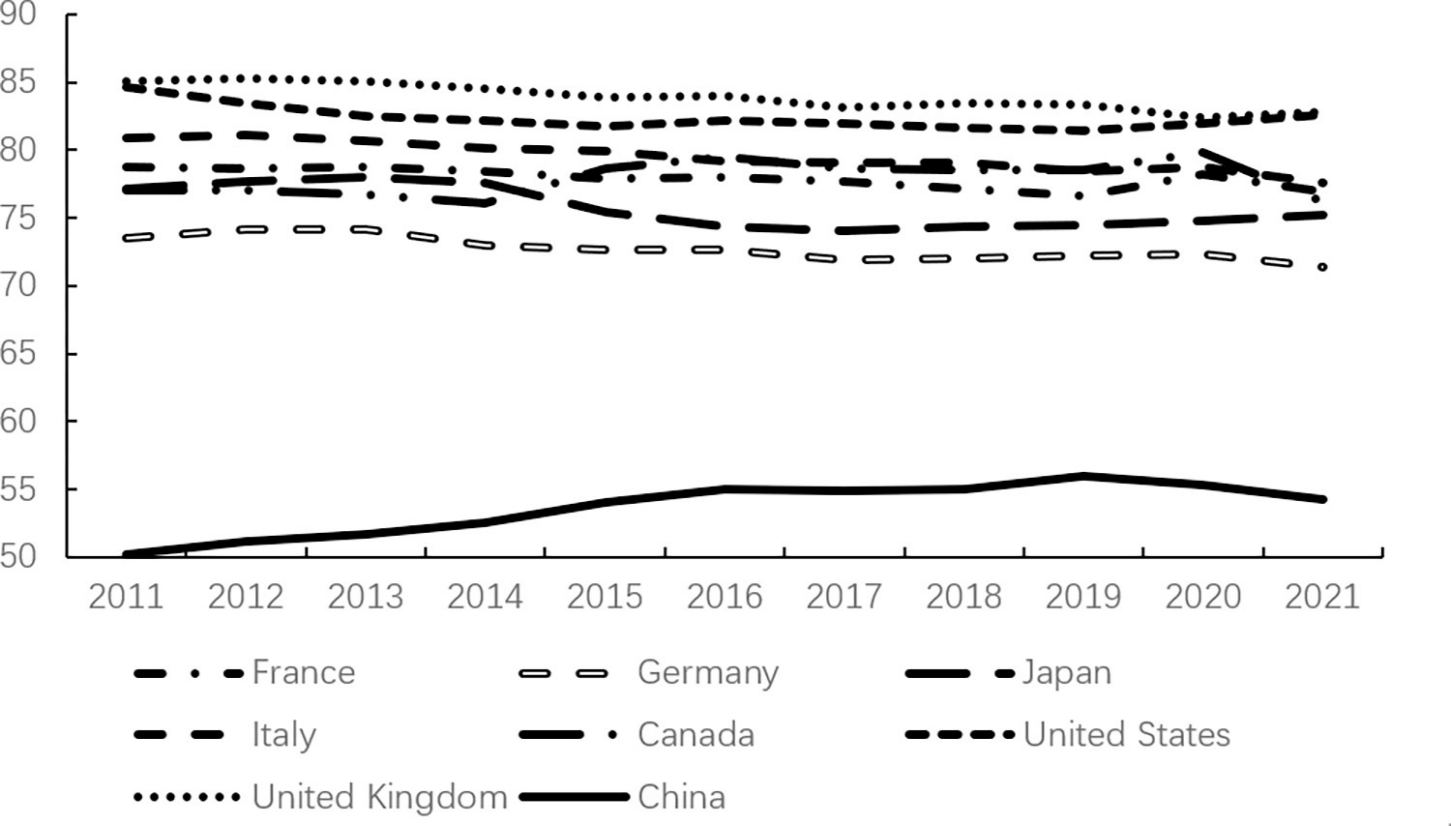
Final consumption expenditure in China and G7 countries (% of GDP).

**Fig 2:**
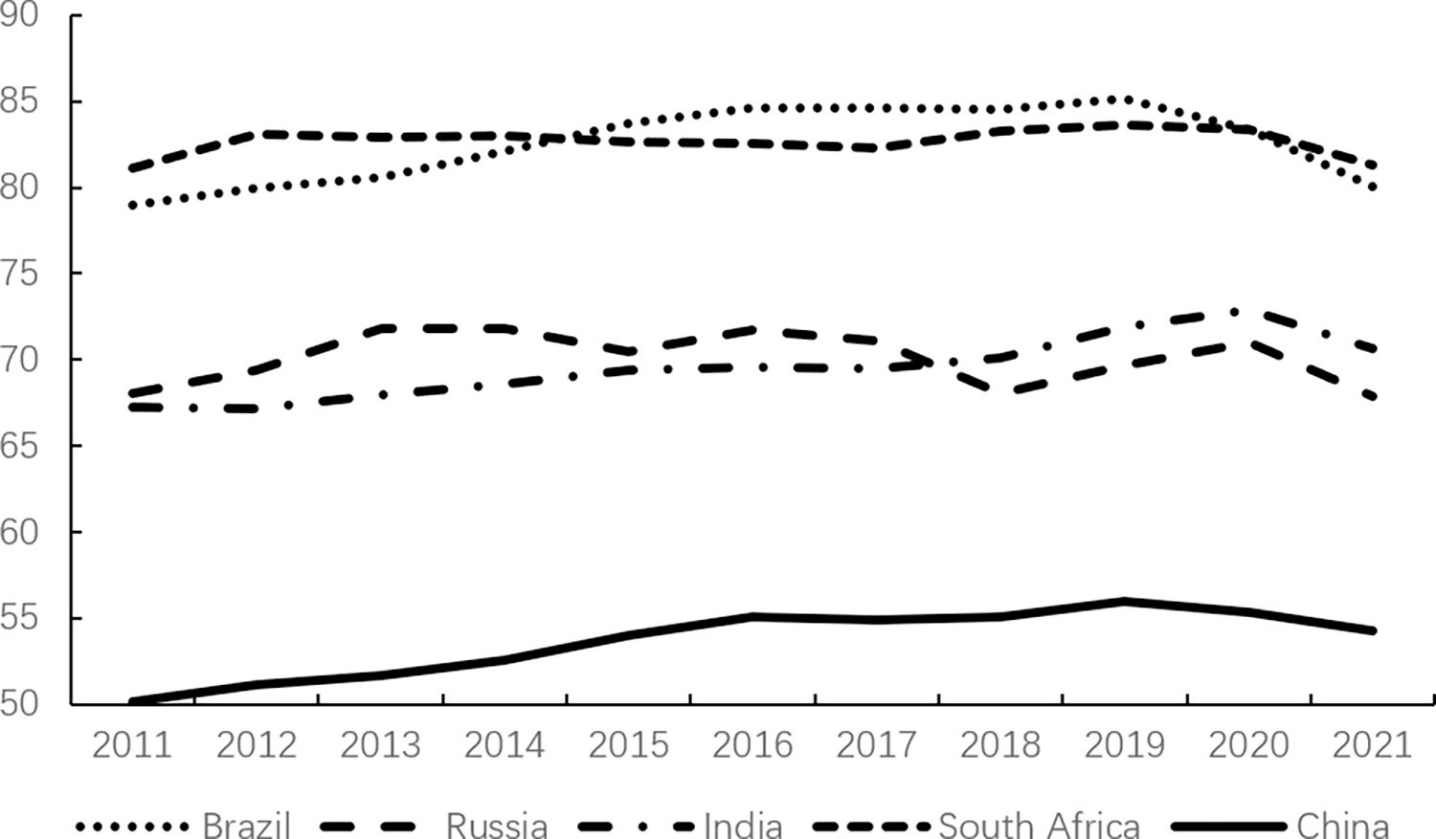
Final consumption expenditure in China and BRICS countries (% of GDP).

**Fig 3:**
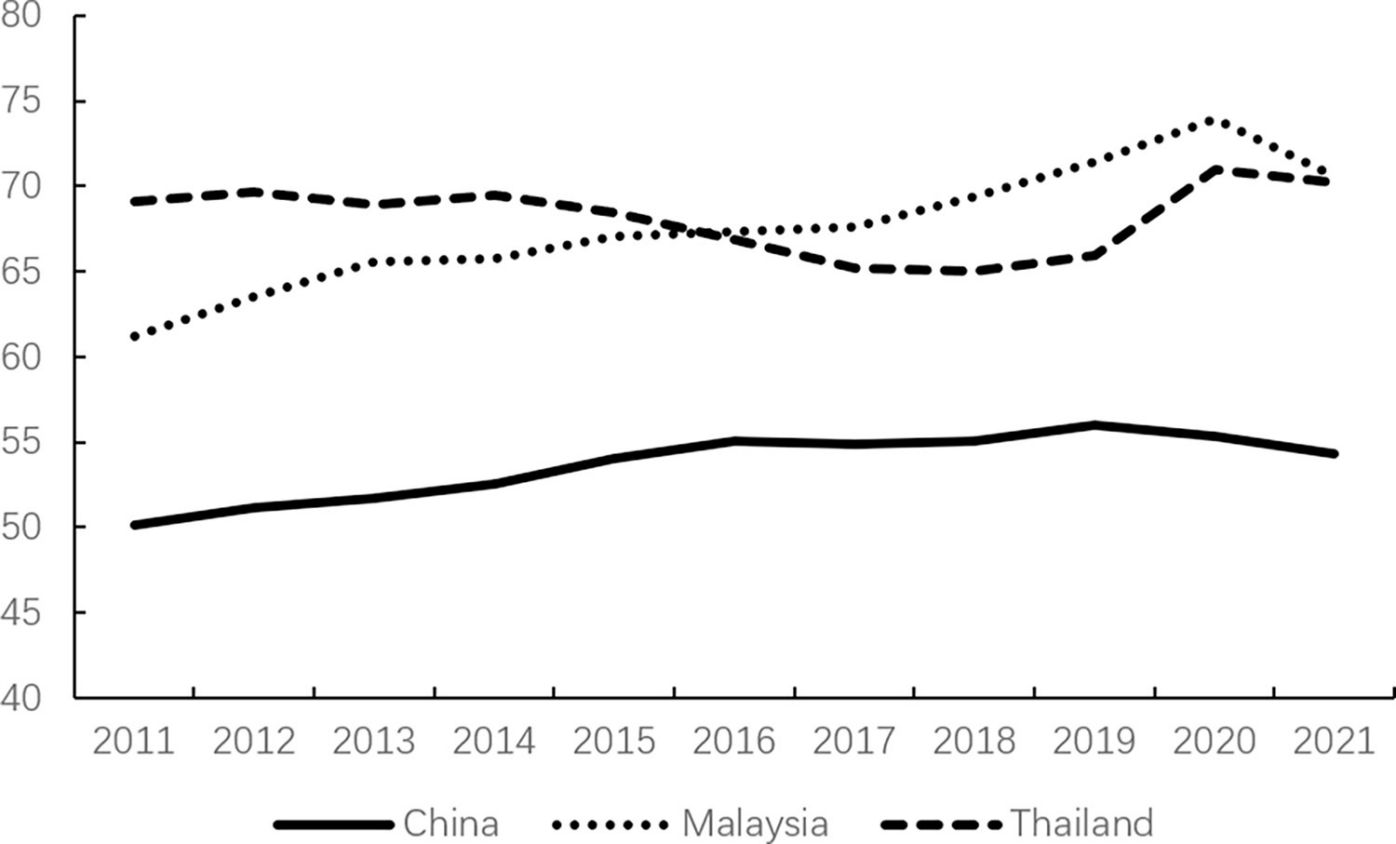
Final consumption expenditure in China, Malaysia, and Thailand (% of GDP).

**Fig 4:**
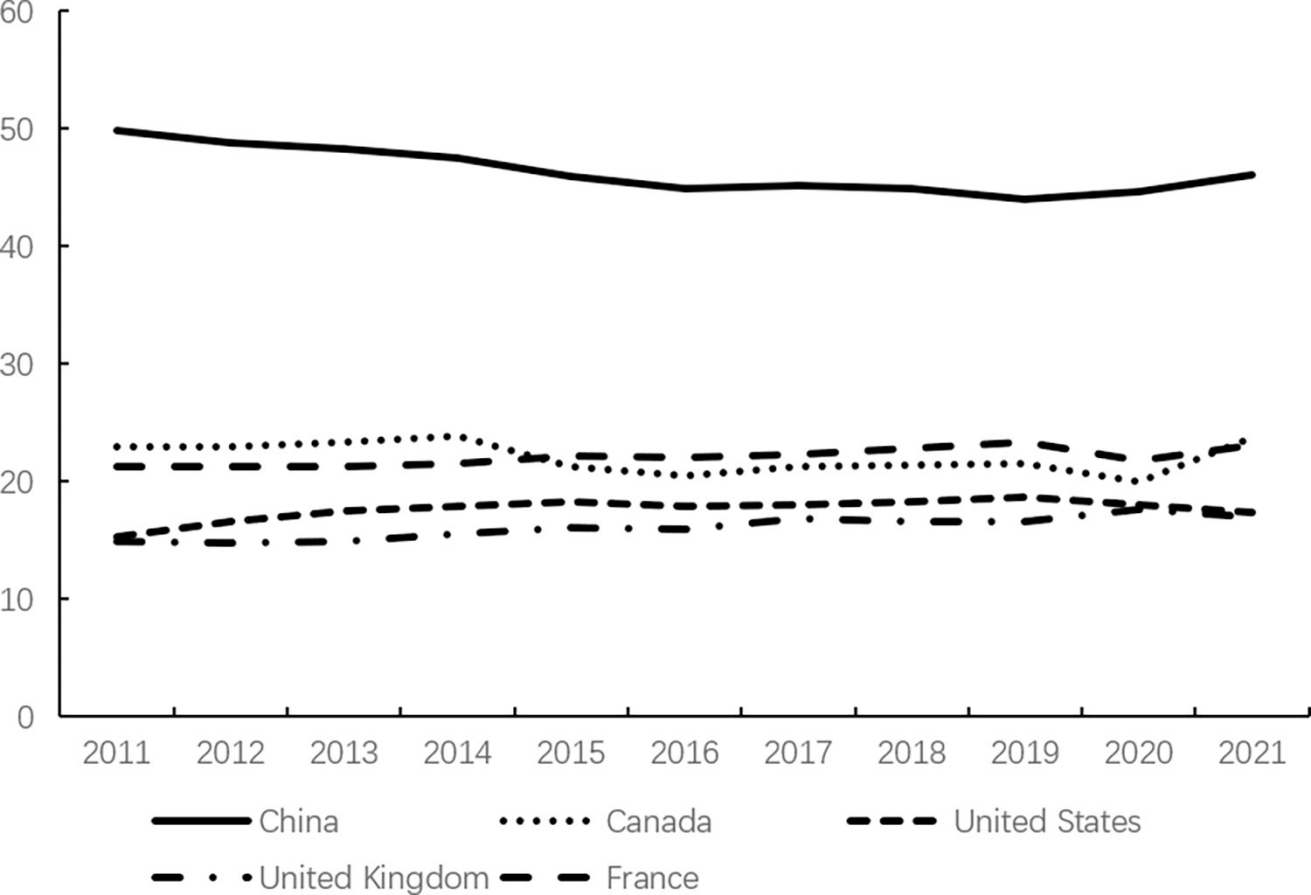
Gross domestic savings (% of GDP).

Consumption, as one of the core factors driving economic growth, plays a crucial role in the continuous and stable development of the economy and society through the evolution of its scale and structure. However, consumption expenditure in China has remained relatively low. According to data from the World Bank, as illustrated in Figs 1–3, China’s consumption expenditure is lower than that of the G7 countries and ranks the lowest among the BRICS countries, even falling behind developing countries in the Asia-Pacific region such as Malaysia and Thailand. Meanwhile, in China, savings account for approximately half of the country’s GDP (Fig 4); whereas in developed countries, household savings typically constitute around 20% of GDP [13].

Currently, scholars have conducted numerous studies on the development of digital financial inclusion and the upgrading of consumption structure. Yue et al. (2022) demonstrate that the widespread use of digital finance enhances credit market participation, thereby fostering household borrowing and elevating consumption levels through altered marginal propensity to consume [14]. Digital financial inclusion will use its digital and precise characteristics to precisely improve the consumption structure of rural residents [4]. Digital finance has significant fairness effects in reducing poverty, increasing consumption, and promoting financial asset holding [15]. Hashemizadeh et al. (2023) reveal a positive correlation between digital financial inclusion, information and communication technology, population growth, and non-financial investments in OECD countries [16].

Previous studies have primarily focused on the relationship between digital financial inclusion and consumption, or digital financial inclusion and population growth; however, the impact of digital financial inclusion on the upgrading of urban and rural consumption structures, as well as the moderating effects of population structure in this process, remains unexplored. Against the backdrop of the increasingly severe trend of population aging, a thorough exploration of the potential impact of digital financial inclusion on consumption scale and structure, as well as the moderating effect of population structure in this process, not only contributes to enriching and improving relevant theoretical systems, but also provides scientific decision-making basis for policymakers, thus possessing significant theoretical value and practical significance. Furthermore, given that the upgrading of consumption structure has become a crucial driving force for promoting high-quality economic development, and digital financial inclusion and population structure changes jointly influence the adjustment and optimization of consumption structure, a systematic analysis of their interaction mechanisms is of great practical significance for deeply understanding the inherent logic of consumption structure upgrading, guiding financial service and product innovation, and promoting the harmonious and sustainable development of the economy and society. This paper establishes fixed effect model to test the effect of digital financial inclusion on the upgrading of consumption structure in urban and rural, and further explores the moderating effect of population structure.

The rest of this paper is as follows. Section 2 is a theoretical analysis and research hypotheses. The methodology is elucidated in Section 3. Section 4 is the empirical results and analysis. Section 5 is the discussion. Conclusions and future prospects are presented in Section 6.

## 2. Theoretical analysis and research hypotheses

Digital financial inclusion enhances consumers’ wealth management capabilities: By digital financial inclusion, consumers can better understand financial products and services, thus managing their wealth more effectively. This can help consumers better plan their consumption plans. Overall, the digital financial inclusion can affect resident households’ financial decisions and behaviour.

The widespread use of digital finance increases credit market participation. And increased participation to credit markets, in turn, stimulates household borrowing and leads to a higher level of consumption by changing the marginal propensity to consume [14]. Digital financial inclusion can significantly promote the improvement of residents’ consumption level through the mediating mechanism of increasing residents’ income, facilitating payment and optimizing industrial structure, and the effect is more obvious in the eastern coastal regions and non-complex terrain regions [17]. As a key contributing factor to economic growth and investment opportunity, financial inclusion increases consumer spending and consequently business development [18]. Digital payments (value) positively affect consumers’ current situation and future expectations [19]. Financial inclusion increased dietary diversity and food consumption by 12 and 14 percent, respectively. It indicates that pro‐rich advantages in financial inclusion [20]. Ait and Gomis (2021) propose a monetary model with endogenous credit market participation to study the impact of financial inclusion on inequality and welfare. They find that consumption inequality results from differences in agents’ decision to access financial services. This heterogeneity generates a pecuniary externality, potentially resulting in some agents overconsuming [21]. Feng and Du (2023) demonstrate digital financial inclusion will significantly weaken the positive impact of financial knowledge on the scale and structure of households’ consumption [22]. Financial inclusion increases access to the formal banking system and financial markets, and allows households to use more appropriate financial instruments, which should enable them to diversify consumption, disrupting the connection to idiosyncratic income shocks [23].

Based on the above analysis, this paper puts forward the hypothesis:

H1. Digital financial inclusion will expand the scale of consumption.
H2. Digital financial inclusion can promote the upgrading of consumption structure.

The strength of the role of digital financial inclusion in residents’ consumption upgrading will also change due to changes in the external environment. In the digital era, new financial technologies and big data are accelerating the development of financial transactions [24]. With the rapid popularization of mobile Internet, mobile offline third-party payment has penetrated into all aspects of daily transactions, and has a profound impact on people’s consumption habits, payment methods and original financial management concepts [25]. According to the findings of the research, the elements that have the most impact on a person’s ability to participate in the financial system include their level of income, demographic category, gender, marital status, age, and educational attainment [26].

Digital financial inclusion exerts a positive effect on urban population growth [27]. Theories reveal that age influences individuals’ saving and consumption behaviour [28]. The level, as well as the structure of consumption, is influenced by the age structure of the population. Population aging may imply smaller household sizes, which will change the consumption structure. Fair and Dominguez (1991) show that per capita consumption declines with the share of middle-aged individuals, a result which is argued to be in line with life-cycle theory that predicts the bulk of saving to occur among those in prime age [29]. Consumer preference shifts and different attitudes towards consumption among the generations determine additional effects. Because consumption decisions are influenced by not only personal characteristics but also social trends and environment, it is one-sided to analyze the impact of one single factor on the phenomenon of consumption [30].

Based on the above analysis, this paper puts forward the hypothesis:

H3. population structure will regulate the promotional effect of digital financial inclusion on the upgrading of consumption structure.

Gender is an important factor when analyzing the effect of digital financial inclusion on the upgrading of consumption structure. The profiles of female consumers and male consumers differ substantially. For example, men more conspicuous consumption for the purpose of interpersonal mediation and ostentation [31]. Women are significantly more involved in conspicuously buying high fashion clothing items than men to communicate status and identity to others [32]. Thus, in view of these gender differences, it is conceivable that the relationship between the digital financial inclusion and the upgrading of consumption structure may be influenced by gender.

Based on this, this paper proposes the hypothesis:

H4. Compared to females, males will demonstrate greater the promotional effect of digital financial inclusion on the upgrading of consumption structure.

## 3. Study design

### 3.1 Model construction

In this section, we outline the empirical design of the study, detailing the research methodology, and data sources utilized to examine the relationship between digital financial inclusion and consumption upgrades.

To examine the relationship between digital financial inclusion and the scale of consumption, as well as to verify Hypothesis H1 of this study, we utilize the following basic model [33,34].


$$\ln\;Total_{it}=\alpha_{0}+\alpha_{1}\cdot \ln\;DFI_{it}+\alpha_{3}\cdot Control_{it}+\varepsilon_{it}$$ (1)

*Total* denotes the scale of consumption; *DFI* is the digital financial inclusion. *Control* denotes a vector of additional factors influencing consumption; and *ε*_*it*_ is the random disturbance term. And the subscript *i* denotes city, *t* indicating time.

In order to test the effect of digital financial inclusion on the upgrading of consumption structure, as well as to verify Hypothesis H2 of this study, we have established a fixed-effects regression model, presented as Eq (2):

$$CSU_{it}=\beta_{0}+\beta_{1}\cdot \ln\;DFI_{it}+\beta_{3}\cdot Control_{it}+\varepsilon_{it}$$ (2)

*CSU*_*it*_ denotes the upgrading of consumption structure, and the subscript *i* denotes city, *t* indicating time. Among them, if the coefficient *β*_1_>0, it indicates that digital financial inclusion can promote the upgrading of consumption structure.

To examine the significant role of population structure in the process by which digital financial inclusion (DFI) empowers the upgrading of consumption structures (CSU), and to test the hypotheses H3 and H4 of this study, we introduce population structure as a moderating variable into the benchmark model. The model is specified as follows:

$$CSU_{it}=\gamma_{0}+\gamma_{1}\cdot \ln\;DFI_{it}+\gamma_{2}\cdot Child_{it}+\gamma_{3}\cdot \ln\;DFI\times Child_{it}+\gamma_{4}\cdot Control_{it}+\varepsilon_{it}$$ (3)

$$CSU_{it}=\gamma_{0}+\gamma_{1}\cdot \ln\;DFI_{it}+\gamma_{2}\cdot Old_{it}+\gamma_{3}\cdot \ln\;DFI\times Old_{it}+\gamma_{4}\cdot Control_{it}+\varepsilon_{it}$$ (4)

$$CSU_{it}=\gamma_{0}+\gamma_{1}\cdot \ln\;DFI_{it}+\gamma_{2}\cdot Sex_{it}+\gamma_{3}\cdot \ln\;DFI\times Sex_{it}+\gamma_{4}\cdot Control_{it}+\varepsilon_{it}$$ (5)

Among them, if the interaction term coefficient γ3 is significantly positive, it indicates that population structure in the process of digital financial inclusion development can better promote the upgrading of consumption structure. If the interaction coefficient is negative, it means that the population structure may worsen the local financial environment, which will have a negative impact on the upgrading of consumption structure.

### 3.2 Variables

#### 3.2.1 Explained variables

To examine the relationship between digital financial inclusion and the scale of consumption, as well as to verify Hypothesis H1 of this study. To quantify changes in consumption scale, we utilize the logarithm of total consumption expenditure per capita [35].

There are two primary approaches to measure the upgrading of consumption structure: the first involves segmenting consumption into subsistence and developmental hedonic consumption and employing their ratio as a proxy for consumption upgrading [36]; the second approach focuses on the proportion of developmental hedonic consumption expenditure within total consumption expenditure [37,38]. Following Shi (2019) [34], we measure consumption structure upgrading as the second approach, to test the effect of digital financial inclusion on the upgrading of consumption structure. We exclude housing expenditure from total consumption, as its significant share can lead to crowding-out effects on other non-residential consumption [39]. The specific calculation method is as follows [40]:

The upgrading of consumption structure (CSU) = (expenditure on clothing + expenditure on daily necessities and services + expenditure on transportation and communication + expenditure on education, culture, and entertainment + expenditure on healthcare) / (total consumption—housing expenditure).

#### 3.2.2 Core explanatory variables

The core explanatory variable in our analysis is Digital Financial Inclusion (DFI). Following the methodology of Guo et al. (2019), we measure DFI using the Peking University Digital Financial Inclusion Index [30]. The Digital Financial Inclusion (DFI) index is a multi-dimensional construct that encompasses factors related to the accessibility and utilization of digital financial services [41]. It assesses the degree to which individuals and businesses within a region can access digital banking, mobile payment systems, online lending platforms, and other digital financial instruments [42,43]. Additionally, the index takes into account the integration of digital finance within the broader financial ecosystem, including the adoption of fintech innovations and the development of digital infrastructure [44]. The measurement process involves compiling data from a variety of sources, such as financial institutions, government agencies, and technology providers [45].

#### 3.2.3 Control variables

The control variables include child dependency ratio (*Child*), old-age dependency ratio (*Old*), sex ratio (*Sex*), per capita disposable income (*lnIncome*), urban economy (*Economy*), real interest rate (*Rate*), Gross Domestic Product per capita (*Per capita GDP*), the basic medical insurance (*Medical*), unemployment insurance (*Unemployment*), university students (*lnStudent*), educational expenditure (*lnEducation*), internet users (*lnInternet*), governments’ behavior (*Government*). And control for the time (time) and city (city) fixed effects. See Table 1 for variable definitions.

**Table 1:** Variable descriptions.

variable	Variable name	Variable symbol	Define
**Explained variables**	The scale of consumption	*lnTotal*	ln (Consumption spending)
	The upgrading of consumption structure	*CSU*	The proportion of developmental hedonic consumption expenditure within total consumption expenditure
**Core explanatory variables**	Digital financial inclusion	*lnDFI*	Peking University Digital Financial Inclusion Index Overall Index
**Control variables**	Child dependency ratio	*Child*	Number of child persons (0–14) / Number of working persons (15–64)
	Old-age dependency ratio	*Old*	Number of older persons (65+) / Number of working persons (15–64)
	Sex ratio	*Sex*	The number of men divided by the number of women in each prefecture-level city
	Per capita disposable income	*lnIncome*	The logarithm of the per capita disposable income is used
	Urban economy	*Economy*	RMB loan in finance institutions / GDP
	Real interest rate	*Rate*	Real interest rate
	Gross Domestic Product per capita	*Per capita GDP*	GDP / the number of permanent residents
	The basic medical insurance	*Medical*	Share of basic medical insurance enrolment to total
	Unemployment insurance	*Unemployment*	Share of unemployment insurance enrolment to total
	university students	*lnStudent*	ln (university students)
	Educational expenditure	*lnEducation*	ln (Educational expenditure)
	Internet users	*lnInternet*	ln (Internet users)
	Governments’ spending behavior	*Government*	financial expenditures / GDP

### 3.3 Data source

Digital financial inclusion is increasingly recognized as a pivotal factor in economic transformation. The influence of population structure, a critical driver in achieving consumption upgrading objectives, is also significant and cannot be overlooked. Given the scarcity of data in certain cities and the challenges in acquiring specific variables before 2013, coupled with the fact that data on digital financial inclusion post-2021 has not yet been released, and considering the substantial economic disruption caused by the COVID-19 pandemic in 2020, this study employs a fixed-effects model to examine the impact of digital financial inclusion on the upgrading of consumption structures in both urban and rural areas across 248 prefecture-level cities from 2013 to 2019. The data sources utilized in this analysis include digital financial inclusion metrics from the Peking University Digital Finance Research Center (https://idf.pku.edu.cn/), the China Urban Statistical Yearbook, and statistical yearbooks from individual prefecture-level cities.

We divide the data into two samples: urban and rural. Due to significant differences in lifestyle, mindset, and various aspects between urban and rural residents, and also significant variations in sample sizes between urban and rural areas. Considering only the total national consumption expenditure does not fully reflect the impact of digital financial inclusion on consumption structure and is not rigorous enough. In addition, there is a severe lack of data on overall consumer expenditure categories.

The descriptive statistical results of the main variables are shown in Tables 2 and 3.

**Table 2:** Descriptive statistics of relevant variables in towns.

	N	Mean	Std	Min	Max
**lnTotal**	1617	9.828	0.246	9.334	10.471
**CSU**	1617	0.615	0.061	0.461	0.739
**lnDFI**	1617	5.266	0.232	4.670	5.670
**lnIncome**	1617	10.254	0.236	9.808	10.886
**lnEducation**	1614	4.052	0.756	2.149	6.521
**lnStudent**	1617	1.463	1.294	-1.433	4.477
**lnInternet**	1614	4.371	0.874	2.303	6.510
**Government**	1614	0.202	0.113	0.055	2.061
**Medical**	1617	0.254	0.265	0.038	1.545
**Unemployment**	1613	0.141	0.222	0.015	2.836
**Rate**	1617	0.037	0.055	-0.173	0.257
**per capita GDP**	1614	5.280	2.939	1.266	16.501
**Economic**	1617	1.153	0.837	0.340	4.530
**Child**	1617	23.915	5.784	12.517	38.700
**Old**	1617	16.850	5.036	8.070	32.647
**Sex**	1617	104.907	4.395	96.199	118.312

**Table 3:** Descriptive statistics of relevant variables in rural areas.

	N	Mean	Std	Min	Max
**lnTotal**	1545	9.144	0.318	8.420	9.910
**CSU**	1543	0.580	0.077	0.400	0.738
**lnDFI**	1545	5.266	0.234	4.670	5.670
**lnIncome**	1545	9.431	0.335	8.570	10.280
**lnEducation**	1542	4.076	0.746	2.109	6.521
**lnStudent**	1545	1.491	1.317	-1.457	4.488
**lnInternet**	1542	4.381	0.886	2.197	6.519
**Government**	1542	0.198	0.113	0.055	2.061
**Medical**	1545	0.245	0.246	0.037	1.252
**Unemployment**	1541	0.133	0.176	0.015	2.113
**Rate**	1545	0.041	0.035	-0.094	0.148
**per capita GDP**	1545	5.338	2.920	1.207	16.132
**Economic**	1545	1.128	0.819	0.324	4.310
**Child**	1545	24.250	5.596	12.494	38.800
**Old**	1545	16.569	4.833	8.070	32.470
**Sex**	1545	105.072	4.433	96.012	118.416

It is clear that the average of residents’ consumption expenditure in urban areas is higher than that in rural areas, and the difference in the extreme values of consumption levels in rural areas is larger than in urban areas, indicating that the disparity in rural consumption levels is more extreme. CSU is higher in urban areas, while it is lower in rural areas, which may reflect the different consumption habits and demand levels among residents in different regions.

Moreover, both urban and rural areas are facing the problem of an aging population, but the specific degree may vary. In some regions, the degree of aging in prefecture-level cities is already high, which may be reflected in both urban and rural areas. In terms of gender ratio, in some prefecture-level cities, the number of male population is significantly higher than that of female population. This phenomenon may exist in both urban and rural areas, but the specific differences need further analysis.

## 4. Empirical results and analysis

### 4.1 Regression analysis of benchmark model

#### 4.1.1 Benchmark regression results of consumption scale

The impact of digital financial inclusion on the scale of consumption in Table 4. From the empirical results, digital financial inclusion is significantly positive at the 5% significance level, indicating that digital financial inclusion can significantly expand the scale of consumption, and Hypothesis 1 is tested. Column (4): The regression coefficient of digital financial inclusion is 0.113. When the level of digital financial inclusion increases by 1%, the consumption scale of urban residents will increase by 0.113%. Column (8): The regression coefficient of digital financial inclusion is 0.204. When the level of digital financial inclusion increases by 1%, the consumption scale of rural residents will increase by 0.204%. The possible reason is that there are more severe constraints on mobility in rural areas. digital financial inclusion breaks through the limitations of time, geography, and hardware facilities, and penetrates into rural areas in a more efficient and convenient way, which is more in line with the borrowing needs of rural areas. Therefore, in rural areas, inclusive finance can alleviate financing constraints and increase financing channels, in order to better promote consumption expenditure.

**Table 4:** Benchmark regression results of consumption scale.

	Urban				Rural			
	(1)	(2)	(3)	(4)	(5)	(6)	(7)	(8)
**lnDFI**	0.410*** (9.68)	0.388*** (8.38)	0.303*** (6.51)	0.113** (2.27)	0.359*** (6.20)	0.402*** (6.24)	0.281*** (4.30)	0.204*** (2.80)
**lnIncome**	0.789*** (38.75)	0.804*** (37.99)	0.735*** (32.70)	0.648*** (25.89)	0.682*** (35.57)	0.676*** (34.31)	0.629*** (30.54)	0.586*** (27.28)
**lnEducation**		-0.014* (-1.82)	-0.012 (-1.58)	-0.017** (-2.30)		0.031*** (2.82)	0.024** (2.31)	0.010 (0.94)

**lnStudent**		0.011*** (3.22)	0.012*** (3.53)	0.003 (0.81)		-0.025*** (-5.13)	-0.027*** (-5.70)	-0.037*** (-7.74)
**lnInternet**		-0.003 (-0.32)	-0.008 (-0.99)	0.010 (1.25)		0.013 (1.17)	0.015 (1.35)	0.034*** (3.00)

**Government**		0.084*** (3.20)	0.012 (0.45)	-0.034 (-1.09)		0.028 (0.75)	-0.047 (-1.23)	-0.063 (-1.50)
**Medical**			0.096*** (4.70)	0.062*** (3.05)			0.073** (2.55)	0.029 (1.00)
**Unemployment**			0.035 (1.64)	-0.008 (-0.35)			0.125*** (3.55)	0.085** (2.48)
**Rate**				-0.146*** (-3.02)				-1.431*** (-10.55)

**per capita GDP**				0.013*** (7.27)				0.012*** (5.47)
**Economic**				0.031*** (6.26)				0.015** (2.19)
**Constant**	-0.279** (-2.08)	-0.294* (-1.73)	0.823*** (3.83)	2.524*** (8.68)	0.807*** (4.36)	0.520** (2.27)	1.552*** (5.74)	2.284*** (7.10)
**Observations**	1617	1614	1613	1612	1545	1542	1541	1541
**R-squared**	0.812	0.814	0.822	0.832	0.783	0.793	0.802	0.819
**City-fixed**	YES	YES	YES	YES	YES	YES	YES	YES
**Time-fixed**	YES	YES	YES	YES	YES	YES	YES	YES

Note

***, **, * represent the 1%, 5%, and 10% significance levels, respectively, as below.

#### 4.1.2 Baseline regression of consumption structure

Table 5 shows the regression results based on Formula (2). The results show that the coefficient of digital financial inclusion on consumption structure is significantly positive at the 5% level. The regression coefficient of digital financial inclusion on the consumption structure of urban residents is 0.044, while the regression coefficient on the rural is 0.117, which shows that digital financial inclusion has a positive effect on the upgrading of consumption structure. It indicates that digital financial inclusion can promote the upgrading of consumption structure. The assumption of H2 has been verified. The regression coefficient of inclusive finance in rural areas is relatively high, which may also reflect the more urgent demand for digital financial inclusion by rural residents. Due to the relatively low level of economic development in rural areas, digital financial inclusion play a more significant role in promoting rural economic development and improving the living standards of farmers.

**Table 5:** The regression results of consumption structure.

	Urban				Rural			
	(1)	(2)	(3)	(4)	(5)	(6)	(7)	(8)
**lnDFI**	0.038** (2.25)	0.040** (2.31)	0.040** (2.29)	0.044** (2.49)	0.114*** (5.28)	0.114*** (5.23)	0.115*** (5.18)	0.117*** (5.14)
**lnIncome**	0.035** (2.12)	0.034** (2.05)	0.033** (1.98)	0.029* (1.70)	0.045*** (2.92)	0.047*** (3.00)	0.046*** (2.94)	0.047*** (2.96)
**lnEducation**	0.018*** (3.77)	0.021*** (4.34)	0.021*** (4.43)	0.021*** (4.11)	0.008 (1.28)	0.012** (2.01)	0.012* (1.84)	0.012* (1.88)
**lnStudent**		0.007*** (2.74)	0.007*** (2.75)	0.007*** (2.85)		0.003 (1.04)	0.003 (1.07)	0.003 (1.07)

**lnInternet**		-0.006** (-2.32)	-0.006** (-2.37)	-0.006** (-2.29)		-0.003 (-1.01)	-0.003 (-1.12)	-0.003 (-1.13)

**Government**		-0.029*** (-2.79)	-0.029*** (-2.79)	-0.028*** (-2.58)		-0.045*** (-3.36)	-0.043*** (-3.25)	-0.045*** (-3.25)
**Medical**			0.006 (1.16)	0.005 (0.87)			0.012 (1.63)	0.011 (1.50)
**Unemployment**			0.011 (0.64)	0.004 (0.24)			-0.030 (-0.71)	-0.031 (-0.73)
**Rate**			0.001 (0.07)	0.002 (0.21)			-0.169* (-1.88)	-0.167* (-1.85)
**Per capita GDP**				0.001 (1.15)				-0.000 (-0.23)
**Economic**				0.004 (1.32)				0.001 (0.31)
**Constant**	-0.045 (-0.24)	-0.034 (-0.18)	-0.027 (-0.14)	-0.015 (-0.08)	-0.483*** (-2.95)	-0.498*** (-3.03)	-0.490*** (-2.96)	-0.506*** (-2.98)
**Observations**	1,614	1,614	1,613	1,612	1,540	1,540	1,539	1,539
**R-squared**	0.687	0.692	0.692	0.693	0.671	0.674	0.676	0.676
**City-fixed**	YES	YES	YES	YES	YES	YES	YES	YES
**Time-fixed**	YES	YES	YES	YES	YES	YES	YES	YES

#### 4.1.3 Moderating effects of population structure

Table 6 shows the regression results based on Formulas (3), (4) and (5). Columns (2) and (6) report the regression results of the interaction term between digital financial inclusion and child dependency ratio. It can be seen that the interaction between digital financial inclusion and child dependency ratio is significantly positive at the 1% significance level, indicating that child dependency ratio has a positive moderating effect on digital financial inclusion-driven the upgrading of consumption structure. One possible reason is that, with the development of the times, the shift in consumer attitudes, and the increase in income levels. Residents are more inclined to increase consumption expenditure for their children, based on the premise of not lacking material resources. Columns (3) and (7) report the regression results of the interaction term between digital financial inclusion and old-age dependency ratio. Although the interaction term coefficient between digital financial inclusion and old-age dependency ratio is negative, it does not pass the test of significance. Columns (4) and (8) report the regression results of the interaction term between digital financial inclusion and sex ratio. At the significance effect of 1%, the interaction between digital financial inclusion and sex ratio is significantly positive, indicating that sex ratio has a positive moderating effect on digital financial inclusion-driven the upgrading of consumption structure. The higher the proportion of males, the stronger the positive impact of digital financial inclusion on the upgrading of consumption structure. Hypothesis 3 and 4 are proved.

**Table 6:** Regression results of population structure moderating effects.

	Urban				Rural			
	(1)	(2)	(3)	(4)	(5)	(6)	(7)	(8)
**lnDFI**	0.044** (2.49)	0.020 (1.09)	0.049*** (2.66)	0.030 (1.64)	0.117*** (5.14)	0.065*** (2.83)	0.120*** (5.17)	0.105*** (4.48)
**Child**		0.023 (0.47)				-0.012 (-0.20)		
**lnDFI⊆ Child**		0.211*** (4.38)				0.543*** (8.83)		
**Old**			0.092* (1.95)				0.096 (1.55)	
**lnDFI⊆ Old**			-0.015 (-0.23)				-0.009 (-0.12)	
**Sex**				0.014 (0.28)				-0.060 (-1.02)
**lnDFI⊆ Sex**				0.178*** (3.12)				0.231*** (3.17)
**lnIncome**	0.029* (1.70)	0.024 (1.42)	0.031* (1.80)	0.028 (1.63)	0.047*** (2.96)	0.014 (0.90)	0.054*** (3.31)	0.032* (1.94)
**lnEducation**	0.021*** (4.11)	0.014*** (2.62)	0.022*** (4.30)	0.020*** (3.90)	0.012* (1.88)	-0.004 (-0.65)	0.013** (1.97)	0.011* (1.72)
**lnStudents**	0.007*** (2.85)	0.006** (2.25)	0.008*** (3.03)	0.006** (2.50)	0.003 (1.07)	0.001 (0.17)	0.004 (1.18)	0.003 (0.85)
**lnInternet**	-0.006** (-2.29)	-0.006** (-2.56)	-0.005** (-2.01)	-0.006** (-2.53)	-0.003 (-1.13)	-0.005 (-1.58)	-0.003 (-1.03)	-0.004 (-1.17)
**Government**	-0.028*** (-2.58)	-0.019* (-1.75)	-0.030*** (-2.70)	-0.026** (-2.37)	-0.045*** (-3.25)	-0.022 (-1.61)	-0.046*** (-3.37)	-0.041*** (-3.01)
**Medical**	0.005 (0.87)	0.004 (0.67)	0.003 (0.63)	0.005 (0.89)	0.011 (1.50)	0.007 (1.01)	0.010 (1.26)	0.011 (1.52)
**Unemployment**	0.004 (0.24)	0.002 (0.13)	0.002 (0.13)	0.003 (0.19)	-0.031 (-0.73)	-0.012 (-0.30)	-0.039 (-0.89)	-0.020 (-0.46)
**Rate**	0.002 (0.21)	-0.001 (-0.11)	-0.002 (-0.18)	0.005 (0.43)	-0.167* (-1.85)	-0.167* (-1.90)	-0.183** (-2.00)	-0.163* (-1.81)
**per capita GDP**	0.001 (1.15)	0.001 (1.18)	0.001 (1.35)	0.002 (1.58)	-0.000 (-0.23)	0.001 (0.42)	-0.000 (-0.12)	0.000 (0.22)
**Economic**	0.004 (1.32)	0.006* (1.86)	0.004 (1.21)	0.005* (1.79)	0.001 (0.31)	0.004 (1.03)	0.002 (0.41)	0.002 (0.50)
**Constant**	-0.015 (-0.08)	0.176 (0.89)	-0.074 (-0.38)	0.057 (0.28)	-0.506*** (-2.98)	0.108 (0.59)	-0.606*** (-3.37)	-0.249 (-1.32)
**Observations**	1,612	1,612	1,612	1,612	1,539	1,539	1,539	1,539
**R-squared**	0.693	0.698	0.694	0.695	0.676	0.695	0.676	0.678
**City-fixed**	YES	YES	YES	YES	YES	YES	YES	YES
**Time-fixed**	YES	YES	YES	YES	YES	YES	YES	YES

### 4.2 Robustness test

(1) Variable substitution method: replace the explained variable with the consumption upgrade index for regression. The specific calculation method of the consumption upgrade index (Upgrade) is as follows:

Upgrade=1×Low+2×Middle+3×High (6)

Among them, *Low* denotes the proportion of food, tobacco, and alcohol expenditures in total expenditure. *Middle* denotes the proportion of housing expenditure in total expenditure. *High* denotes the proportion of expenditure on clothing, household goods and services, transportation and communication, education, entertainment, and healthcare in total expenditure [46,47]. The results are shown in Table 7.

**Table 7:** Robustness test: Variable substitution.

	Urban				Rural			
	(1)	(2)	(3)	(4)	(5)	(6)	(7)	(8)
**lnDFI**	0.129*** (4.48)	0.087*** (2.92)	0.131*** (4.42)	0.109*** (3.64)	0.136*** (2.86)	0.040 (0.83)	0.141*** (2.90)	0.120** (2.45)
**Child**		0.071 (0.89)				0.027 (0.22)		
**lnDFI⊆ Child**		0.363*** (4.64)				0.991***	0.205	
**Old**			0.158** (2.05)				(1.59) -0.017	
**lnDFI⊆ Old**			-0.054 (-0.53)				(-0.10)	-0.306**
**Sex**				-0.036 (-0.46)				(-2.49) 0.550***
**lnDFI⊆ Sex**				0.310*** (3.34)				(3.62) (3.78)
**Control variables**	YES	YES	YES	YES	YES	YES	YES	YES
**City-fixed**	YES	YES	YES	YES	YES	YES	YES	YES
**Time-fixed**	YES	YES	YES	YES	YES	YES	YES	YES
**Observations**	1,612	1,612	1,612	1,612	1,539	1,539	1,539	1,539
**R-squared**	0.257	0.270	0.259	0.263	0.306	0.338	0.308	0.316

(2) Shrinkage treatment method: In addition to using a 1% truncation treatment, this study also tried truncation treatments of 2%, 3%, and 5%. It was found that the regression results did not significantly differ from those obtained after applying the 1% truncation treatment, thus eliminating the interference of outliers.

(3) Sample shortening method: Alipay began to be widely popularized in 2015, and inclusive financial development represented by Alipay has rapidly progressed. Taking 248 prefecture-level cities between 2015 and 2019 as the research objects, and the original model is used for regression, which still shows that digital financial inclusion can promote the upgrading of consumption structure. The results are shown in Table 8.

**Table 8:** Robustness test results from 2015 to 2019.

	Urban				Rural			
	(1)	(2)	(3)	(4)	(5)	(6)	(7)	(8)
**lnDFI**	0.068** (2.41)	0.021 (0.72)	0.064** (2.21)	0.038 (1.31)	0.126*** (3.72)	0.052 (1.55)	0.124*** (3.61)	0.110*** (3.19)
**Child**		-0.071 (-1.19)				-0.213*** (-3.14)		
**lnDFI⊆ Child**		0.423*** (5.50)				0.846*** (9.03)		
**Old**			0.123* (1.77)				0.059 (0.72)	
**lnDFI⊆ Old**			-0.116 (-1.12)				-0.043 (-0.36)	
**Sex**				-0.092 (-1.55)				-0.158** (-2.27)
**lnDFI⊆ Sex**				0.419*** (4.19)				0.343*** (2.79)
**Control variables**	YES	YES	YES	YES	YES	YES	YES	YES
**City-fixed**	YES	YES	YES	YES	YES	YES	YES	YES
**Time-fixed**	YES	YES	YES	YES	YES	YES	YES	YES
**Observations**	1,237	1,237	1,237	1,237	1,172	1,172	1,172	1,172
**R-squared**	0.401	0.419	0.403	0.412	0.477	0.520	0.477	0.482

A series of robustness tests show that the estimated coefficients of digital financial inclusion are significantly positive, which is consistent with the results of the benchmark regression, indicating that the findings of this paper are somewhat robust.

### 4.3 Endogeneity check

To resolve the problem of endogeneity due to missing variables or reverse causality, we sought asuitable instrumental variables to control the endogeneity of the model [48]. Drawing on the method developed by Zhang (2020), “distance from the prefecture-level city to Hangzhou × average inclusive finance in other prefecture-level cities” was used as the first instrumental variable, and the second instrumental variable is the number of financial professionals (Table 9 presents the results) [49]. The p-values of the Kleibergen-Paaprk LM statistic are all less than 0.05, indicating that the instrumental variable passed the unidentifiable test. The F value of the first stage is greater than the empiric value of 10, which excludes the possibility of a weak instrumental variable. When endogeneity is examined, the beneficial effect of DFI on the upgrading of consumption structure remains significant, which once again proves the validity of the research conclusion.

**Table 9:** Instrumental variable regression results.

First-stage regression			Second-stage regression		
	Urban	Rural		Urban	Rural
Explanatory variable	DFI	DFI	Explanatory variable	C	C
**Distance from the prefecture-level city to Hangzhou × average inclusive finance in other prefecture-level cities**	-243.094*** (-194.08)	-229.198*** (-190.19)	**DFI**	0.036** (1.92)	0.073*** (3.22)
**Financial professionals**	0.408** (2.13)	0.205 (0.98)			
**Control variables**	YES	YES	**Control variables**	YES	YES
**City-fixed**	YES	YES	**City-fixed**	YES	YES
**Time-fixed**	YES	YES	**Time-fixed**	YES	YES
**Observations**	1,605	1,534	**Observations**	1,605	1,532
**R-squared**	0.994	0.995	**R-squared**	0.692	0.676
**Kleibergen-Paaprk LM**	0.000	0.000	**Kleibergen-Paaprk LM**		
**F value**	1.9e+04	1.8e+04	**F value**		

### 4.4 Heterogeneity analysis

#### 4.4.1 Regional heterogeneity analysis

Given the uneven development of cities, the issue of regional heterogeneity needs to be further examined by dividing it into eastern, central, and western regions, with the results in Table 10.

**Table 10:** Heterogeneity results of consumption structure by region.

	Urban			Rural		
	(1)	(2)	(3)	(4)	(5)	(6)
	East	Middle	West	East	Middle	West
**lnDFI**	-0.0459* (0.0252)	0.0780* (0.0430)	0.0633* (0.0325)	0.0264 (0.0327)	0.126*** (0.0466)	0.175*** (0.0450)
**Control variables**	YES	YES	YES	YES	YES	YES
**City-fixed**	YES	YES	YES	YES	YES	YES
**Time-fixed**	YES	YES	YES	YES	YES	YES
**Observations**	598	531	483	544	513	482
**R-squared**	0.808	0.708	0.644	0.789	0.725	0.638

From columns (1) to (3), it can be seen that the development of digital financial inclusion in the central and western regions can significantly promote the consumption upgrade of urban residents. However, in eastern urban areas, digital financial inclusion may inhibit consumption upgrade. Columns (4) to (6) indicate that the development of digital financial inclusion in the eastern, central, and western regions can all promote the consumption upgrade of rural residents. However, the upgrade effect is not significant in the eastern region. The coefficients of DFI in the central and western regions are relatively large, possibly because digital financial inclusion and economic development in these regions are relatively slower, income levels are relatively lower, and they face more financing constraints and have fewer avenues for obtaining financing. Therefore, they have a greater need for digital financial inclusion to alleviate financing constraints. It is necessary for the country to focus on the underdeveloped regions in the central and western areas, as well as rural areas, and introduce corresponding policies to promote the development of digital financial inclusion, ultimately driving the upgrade of the residents’ consumption structure.

#### 4.4.2 Heterogeneity of foreign trade dependence

Against the backdrop of the dual-cycle development pattern, we should not only prioritize domestic circulation as the mainstay but also attach importance to the impact of external circulation on China’s consumption. The degree of dependence on foreign trade, to a certain extent, reflects the status of external circulation [50]. The formula for calculating the degree of dependence on foreign trade is the proportion of total import and export in GDP, and cities with high indices are also referred to as export-oriented cities. Changes in the degree of dependence on foreign trade reflect the market, supply, and participation of foreign countries, effectively representing the shifts in the status of external circulation. Therefore, this paper divides cities at the prefecture-level into four groups based on their degree of dependence on foreign trade, namely, low (≤25%), medium-low (>25% and ≤50%), medium-high (>50% and ≤75%), and high (>75%).

As shown in Table 11 in the group with a low degree of dependence on foreign trade, inclusive financial development significantly promotes consumption upgrading for both urban and rural residents. A possible reason is that in cities with low reliance on foreign trade, domestic consumption dominates, thus accelerating inclusive financial development can better stimulate domestic consumer demand, and promote consumption upgrading. In the medium-low and medium-high dependence groups, inclusive finance also significantly drives the consumption upgrading of rural residents. This is probably because with the increase in foreign trade and the enhancement of openness, residents’ demand for foreign markets, such as imported cars and overseas tourism services, is also increasing, and the development of inclusive finance has accelerated this process, promoting the upgrading of consumption structure.

**Table 11:** The results of heterogeneity of foreign trade dependence.

	Urban				Rural			
Explained variables:C	(1)	(2)	(3)	(4)	(5)	(6)	(7)	(8)
	low	low-medium	medium-high	high	low	low-medium	medium-high	high
**lnDFI**	0.0883** (0.0401)	0.00283 (0.0374)	0.0668 (0.0437)	-0.0344 (0.0353)	0.202*** (0.0520)	0.0828* (0.0497)	0.126** (0.0492)	0.00356 (0.0481)
**Control variables**	YES	YES	YES	YES	YES	YES	YES	YES
**City-fixed**	YES	YES	YES	YES	YES	YES	YES	YES
**Time-fixed**	YES	YES	YES	YES	YES	YES	YES	YES
**Observations**	405	400	406	401	377	393	386	383
**R-squared**	0.679	0.716	0.725	0.756	0.655	0.667	0.758	0.723

## 5. Discussion

The empirical results are as follows. First, digital financial inclusion will expand the scale of consumption [35]. The effect of the digital financial inclusion on the consumption scale of rural residents is more obvious (0.204 > 0.113). digital financial inclusion will use its digital and precise characteristics to precisely improve the consumption structure of rural residents [4]. The development of digital financial inclusion will provide new momentum for the integration of rural industries [51]. By facilitating access to diverse financial services and products for rural residents and enterprises, digital financial inclusion significantly contributes to economic growth and overall rural development [42–43]. Compared to the traditional inclusive finance model, digital financial inclusion demonstrates its unique advantages and potential by offering broader coverage and enhanced service efficiency, thereby significantly promoting the development of the rural economy [52]. Digital financial inclusion serves as a pivotal support for addressing financing challenges faced by small and micro enterprises, as well as the agricultural sector, representing a crucial launchpad for financial backing in rural revitalization efforts [53].

Second, digital financial inclusion is significantly positive at the 5% significance level, indicating that digital financial inclusion can promote the upgrading of consumption structure [35], validating hypothesis H2. Similarly, the effect of the digital financial inclusion on the consumption structure of rural residents is more obvious (0.117 > 0.044). The reason for this may be that, compared to urban areas, traditional financial services in rural areas may not have wide coverage, resulting in lower accessibility to financial services. The popularization of digital financial inclusion can greatly compensate for this deficiency by providing rural residents with more diverse and convenient financial services, thereby significantly improving their consumption structure. By expanding access to financial services, digital finance empowers underserved rural communities, fostering entrepreneurship, income generation, and job creation [54].

Third, the interaction between digital financial inclusion and child dependency ratio is significantly positive at the 1% significance level, indicating that child dependency ratio has a positive moderating effect on digital financial inclusion-driven the upgrading of consumption structure. Although the interaction term coefficient between digital financial inclusion and old-age dependency ratio is negative, it does not pass the test of significance. At the significance effect of 1%, the interaction between digital financial inclusion and sex ratio is significantly positive, indicating that sex ratio has a positive moderating effect on digital financial inclusion-driven the upgrading of consumption structure. Therefore, population structure partially moderating the relationship between digital financial inclusion and the upgrading of consumption structure, supporting hypothesis H3 and H4. This indicates that the population structure may affect family consumption decisions and financial service demands. In the future, with further development and improvement of digital financial inclusion, as well as continuous innovation and optimization of financial service needs for different population groups, the impact of population structure on consumption structure upgrading may become more significant and diversified.

Compared with previous studies, the contributions of this study are as follows. Compared with Guo et al [35], this study takes population structure as a moderating variable to analyze the moderating effects of age structure and gender structure in the impact of digital financial inclusion on consumption structure. Secondly, in order to explore the impact of digital financial inclusion on consumption structure in different regions, compared with Liu (2023) [8], this study divides the sample into urban and rural areas and conducts regression analysis separately to compare the differences in the upgrading of consumption structure between urban and rural areas and their potential influencing factors.

This study carries significant theoretical and practical implications, which are outlined as follows. Firstly, this paper reveals the inherent mechanism of population structure in the upgrading of consumption structure. The aim is to provide an in-depth theoretical explanation for the phenomenon of consumption structure changes against the backdrop of population aging. Secondly, we divide the data into two samples: urban and rural. The aim is to offer targeted policy recommendations to policymakers regarding the future development of digital financial inclusion in both urban and rural areas, in order to promote the popularization and optimization of financial services, and drive the upgrading and balanced development of urban and rural consumption structures.

## 6. Conclusions and future prospects

### 6.1 Conclusions

This study empirically tests the role of population structure in the process of digital financial inclusion empowering the upgrading of consumption structure by taking 248 prefecture-level cities between 2013 and 2019 as the research objects. The results of this study are as follows.

First, digital financial inclusion will expand the scale of consumption. Second, digital financial inclusion can promote the upgrading of consumption structure. Third, population structure will regulate the promotional effect of digital financial inclusion on the upgrading of consumption structure. Specifically, child dependency ratio has a positive moderating effect on digital financial inclusion-driven the upgrading of consumption structure. Moreover, sex ratio has a positive moderating effect on digital financial inclusion-driven the upgrading of consumption structure.

The analysis of regional heterogeneity reveals that digital financial inclusion can facilitate the consumption upgrading of both urban and rural areas in central and western China, whereas in eastern urban areas, digital financial inclusion may hinder the consumption upgrading. Regarding the analysis of heterogeneity in foreign trade dependence, in the group with low dependence, the development of digital financial inclusion significantly promotes the consumption upgrading of urban and rural residents; whereas in the groups with moderate-low and moderate-high dependence, digital financial inclusion notably drives the consumption upgrading of rural residents. The regional heterogeneity analysis and the heterogeneity analysis of foreign trade dependence can provide policymakers with more nuanced evidence, assisting them in designing and adjusting inclusive financial policies based on different regions and varying degrees of foreign trade dependence.

### 6.2 Implications on theory and policy

The theoretical implications of this study lie in its contribution to digital financial inclusion and the upgrading of consumption structure. By empirically demonstrating the positive impact of digital financial inclusion on the upgrading of consumption structure, the study reinforces the importance of population structure as a moderating variable for the upgrading of consumption structure. The findings also underscore the role of rural areas as pivotal units in driving the upgrading of consumption structure, emphasizing the need for comprehensive research at this level

The research findings carry critical policy implications for policymakers and stakeholders engaged in digital financial inclusion development efforts. To accelerate rural revitalization, it is crucial to prioritize the construction of rural digital financial inclusion and develop a robust system that enhances financial access for rural areas. Strengthening rural information infrastructure, expanding internet coverage, and integrating rural basic data will facilitate the development of digital financial inclusion in rural [8].

### 6.3 Limitations

This study exhibits certain limitations that require improvement in future research. Firstly, the sample size is small, and there is an imbalance in samples across different regions. And the fact that data on digital financial inclusion post-2021 has not yet been released. There are limitations in terms of sample size, regional selection, and other aspects. Future studies can include a larger sample size and expand the temporal scope of the research. Secondly, while this study has conducted a preliminary exploration of the relationship between the digital financial inclusion (DFI) and the upgrading of consumption structure, it has not yet delved into the details of its internal operating mechanisms. Specifically, the mechanisms through which the digital financial inclusion impacts the upgrading of consumption structure in urban and rural areas in terms of the breadth of coverage, depth of use, and degree of digitization have not been fully revealed. Therefore, it is necessary for future research to further analyze the specific impact pathways and mechanisms of these dimensions on the upgrading of consumption structure in urban and rural areas, in order to enrich and improve the relevant theoretical framework.
