# Expression of Concern: Digital financial inclusion, population structure, and consumption upgrades: Evidence from China

**DOI:** 10.1371/journal.pone.0342733

**Published:** 2026-02-12

**Authors:** The PLOS One Editors

After this article [1] was published, the following concern was noted:

- The articles cited as References 8, 11, and 43 were retracted before [1] was published due to concerns about potential manipulation of the publication process.

We regret that the issues were not identified prior to the article’s publication.

In light of this concern, the *PLOS One* Editors issue this Expression of Concern. Readers are advised to interpret the article [1] with caution.
